# A Study of the Pathogenesis of Gout1Read at the semi-annual meeting of the Medical Society of the County of Erie, June 13, 1899.

**Published:** 1899-08

**Authors:** Mary Clayton

**Affiliations:** Buffalo, N. Y.


### A STUDY OF THE PATHOGENESIS OF GOUT.[1]

BY MARY CLAYTON, M. D., Buffalo, N. Y.

THAT gout is due to a vice of metabolism, associated with an excess of uric acid in the blood, almost all observers agree, but, as our greatest American authority says, "the rest is theory awaiting confirmation or refutation."

It is this transitional state of the theories as to the causation of gout which led me to select this subject, feeling that a résumé of the most important conclusions reached by recent scientific investigators might not be amiss at this time.

1. Read at the semi-annual meeting of the Medical Society of the County of Erie, June 13, 1899.

About the year of the American war for independence uric acid, which was to cause the evolution and revolution of so many pathological theories, was discovered in the urine by Scheele. Three quarters of a century later Sir Alfred Garrod, a name prominent in the history of uric acid, found it in the blood of patients suffering from gout.

Of the many hypotheses as to the pathology of gout which have been brought forward, I have thought it best to consider only two. First, the nervous theory, which is supported by Sir Dyce Duckworth. He maintains that gout is primarily dependent upon a functional disorder of the nervous system, which causes defects of nutrition, not only by undue formation of uric acid, but also by inhibition of the normal destruction of that substance and lessening of the renal excretory power.

In hereditary gout the toxemia is dependent on the inherited gouty neurosis. In acquired gout the toxemia arises from overloading of the digestive and excretory organs, which, if associated with depression of the nervous system, produces the gouty neurosis. I is quite comprehensible that the morbid blood condition, produced by faulty metabolism could affect the trophic center for joints, which is added by the advocates of this theory to the many centers already located in the medulla oblongata. Duckworth does not deny that uric acid is connected with the production of gout, but gives it a secondary place.

The following points are urged as favorable to this theory : (1) The sudden onset of the attack of acute gout, preceded by a feeling of well-being; (2) the explosive neuroses; (3) the influence of depressing circumstances, physical or mental, upon the disease; (3) the relation of gout to diabetes.

Liveing, Latham and Rolfe hold modifications of this theory.

There is one undeniable advantage about pathological theories which depend upon obscure, vaguely located lesions of the nervous system, viz : if they cannot be clearly proven it is almost impossible to disprove them.

In the second view of the pathogenesis of gout, which I wish to present, uric acid is regarded as the primary cause of the disease. Of course uric acid never exists in the body free, but in the form of urates.

Bruce Jones and later Sir William Roberts have demonstrated that uric acid is diblastic and forms three classes of salts : (1) neutral urates, which do not exist in the human organism and so concern us no further at this time; (2) biurates, in which a metal replaces half the displaceable H; (3) quadriurates, in which a metal replaces one-fourth of the displaceable H of two molecules of uric acid.

The quadriurate appears first in the blood in diseases characterised by an excess of uric acid. This salt is soluble but unstable, and after being in the circulation for a varying length of time, the sodium quadriurate unites with some of the sodium carbonate of the blood to form sodium biurates,—a stable but insoluble salt.

The presence of uric acid in the blood in health is affirmed by Halburton, Hammarstein, Watson, Haig and others. It is denied by Garrod, Klemperer, and after original experimentation by Luff. As to the formation of uric acid, Garrod holds that it is normally produced in the kidneys and when in disease it appears in the blood, it is due to reabsorption from these organs.

Hammarstein believes that uric acid originates chiefly from the nuclein of destroyed leucocytes and therefore it is relatively increased in the newborn, after a meal of proteids, in leucocytosis and leucemia, and by drugs which increase the number of leucocytes.

Sir William Roberts suggests that as uric acid is not needed as a vehicle for the elimination of proteids, urea having taken its place in name, perhaps it is one of the vestigial remains. It is pleasant to think that in the far future it will be unnecessary for the followers of Esculapius to wrestle with the problems connected with uric acid, as according to this view of the case evolution will have settled them.

The two books on my subject which are the most widely read are, I think, those of Haig and Luff. I have tried to give in comparison the most important conclusions reached by these authors as they are generally opposed and give different aspects of the subject. Haig's views are very hard to extract from his rambling book, in which his personal sensations occupy such a large place. Many of his conclusions are based upon insufficient premises. He uses a notoriously inaccurate test for uric acid, which throws discredit upon some of his most important theories. If his views as to pathology were accepted in toto the expression uric acid diathesis would be as much abused as the word idiopathic used to be.

Luff's book is condensed and systematic and from the side of theoretical chemistry he makes out a very good case for his theory. According to Haig some of the uric acid is introduced ready formed in the food and is absorbed unchanged into the blood. Uric acid is also formed from the nitrogen of food and in this process the final stage occurs in the kidneys. The greater quantity of uric acid from these two sources is eliminated by the kidneys, but a small residue is carried over by the renal vein into the general circulation, where it is attracted differently by various organs, which tends to be rendered less soluble and is held back in the liver, spleen and certain fibrous tissues, especially those of the joints, probably because these tissues are less alkaline than the rest of the body. This retained uric acid is dissolved when the alkalinity of the blood is increased and passes into the circulation, where it causes headache, slow, high tension pulse, and the like. When an acid is given or the alkalinity of the blood lowered in any way Haig believes that the headache, etc., are relieved because the uric acid is thrown out of the circulation and driven into the joints, where it causes pricking pains. Haig holds that there is a constant relation of uric acid to urea, eliminated by the kidneys of about 1-35, and upon this fact he bases much of his theory. Just as competent observers, however, have found vastly different proportions between these two substances, and it has come to be generally believed that their ratio varies in different individuals.

Another important tenet of his faith is that the alkalinity of the blood varies inversely as the acidity of the urine, and that acid introduced in food or wine reduces the alkalinity of the blood, drives the uric acid into the joints and produces an attack of acute gout.

Luff's experiments on the other hand, seem to prove that the alkalinity of the blood is not greatly reduced in gout and that increased alkalinity does not make the blood a better solvent for uric acid deposited in the form of sodium biurate, which he considers the materies morbi in gout. There is no relation he claims between the alkalinity of the blood and the acidity of the urine.

Haig believes that it is the retention of the quadriurate, probably in the colloid form, which causes all the evils which he attributes to uric acid, except the chronic arthritis of gout and rheumatism.

Sir Wm. Roberts argues that there is no direct proof that uric acid is a toxic agent and though on the eve of an outbreak of acute gout the fluids of the body must be impregnated with it, there are no signs of poisoning. Also in leucocythemia and severe anemia the blood is frequently charged with sodium quadriurate without the production of toxic symptoms referable to this cause. This argument, it seems to me, rather disposes of the retention theory.

The following is a synopsis of Luff's theory as to pathology of gout. Uric acid is not present in the blood in health. It is formed normally in the kidneys, probably from the conjunction of urea and glycoceine. At first the uric acid is in the form of the quadriurates of sodium, potassium and ammonium, which are normally excreted in the urine. In Luff 's estimation gout is always preceded by some functional or organic affection of the kidneys, which interferes with the excretion of uric acid, and its presence in the blood in gout is due to deficient excretion and reabsorption of the three quadriurates mentioned above. These are soon transformed by union with some of the sodium carbonate of the blood into sodium quadriurate. This substance is not toxic, but after circulating in the blood a certain length of time, varying from hours to days, it is converted into sodium biurate, which being insoluble is deposited in the crystalline form in tissues which are especially predisposed to its deposition, either from having received previous slight injuries or because of their poor vascular supply. Here it acts as a foreign body and sets up irritation and inflammation. Visceral neuroses, thrombosis, and the like, may also be explained by the precipitation of minute crystals of sodium biurate, either in the substance of the organ or in the blood itself. The biurate may deposit so slowly and quietly that no acute symptoms are noticed, but this is unusual, the deposition being usually accompanied by pain and the symptoms of inflammation.

The kidney affection associated with gout may be functional and disappear if the exciting cause is removed, or become organic, in which case the epithelium of the convoluted tubules is affected and the granular or cirrhotic kidney results. Liability to this functional kidney affection constitutes the hereditary factor of gout.

In severe anemia and other blood disorders uric acid is formed within the system from the nuclein of the leucocytes, but in this case the kidneys are sound and rapidly eliminate the uric acid. Thus does Luff bridge across the two horns of the dilemma and gain a secure foundation for his theory.

In lead poisoning both these causes are in operation and the uric acid which is found in the blood is due to the renal condition induced by the disease, and to the leucocytosis of the accompanying anemia.

These various pathological theories lead to different plans of treatment for gout. Haig's panacea is a vegetable diet, because he believes that animal food increases the introduction and formation of uric acid and by causing high acidity prevents free elimination of this substance. The alkaloids of tea, coffee and cocoa act in the same way. Wines by their acidity drive the uric acid from the blood into the joints and so cause an attack of acute gout. He believes the diet should consist of milk, cheese, pulses, bread, corn foods, garden vegetables and fruits.

As to drugs Haig holds that colchicum relieves acute gout by increasing the alkalinity of the blood and thus facilitating the solution and excretion of uric acid. Mercury, lead and zinc form insoluble compounds with uric acid and in his graphic words drive it out of the blood and into the joints. The alkalies and salicylates, phosphate of sodium, piperazin and belladonna increase the excretion of uric acid.

Luff's laboratory experiments with artificial blood serum have demonstrated to his satisfaction that a nitrogenous diet does not necessarily produce an excess of uric acid in the system, as increased elimination may compensate for increased intake. He believes that diminution of the alkalinity of the blood, which could be produced by food could not effect its solvent powers for uric acid or the precipitation of sodium biurate The evil effects of wines are not due to their acidity, but to their effect on the metabolism of the liver, causing more glycoceine to go to the kidney. Sodium chloride hastens the formation and precipitation of sodium biurate and diminishes its solubility.

It is possible that the mineral constituents of meat may diminish the solvency of sodium biurate. The mineral constituents of milk have no effect on the solvency of this substance and those of vegetables increase its solubility and delay the change from the quadriurate to the biurate. The vegetables he has found to be most active in this respect are spinach, Brussel sprouts, French beans, cabbage, turnips and celery. Luff, therefore advocates a rational mixed diet including plenty of these vegetables and a table salt composed of their mineral constituents. As to drugs his experiments show positive and sometimes startling results, but his treatment is very much like that advised by others, though he often explains the action of his remedies in an original manner. The salicylates he unqualifiedly condemns, as they bring an increased amount of glycoceine to the kidneys, which are already incapable of eliminating the normal amount of uric acid.

83 NORWOOD AVENUE.

---

A ST. PETERSBURG (*Lancet Clinic; Med. Times*) physician finds that the application of an ointment of ten parts of ichthyol and eighty parts of lanoline prevents pustulation and abbreviates the cutaneous manifestations in small-pox.

